# Female cancers in adolescent and young adult women: global inequities and 2050 burden estimates from GLOBOCAN 2022

**DOI:** 10.3389/fonc.2026.1896950

**Published:** 2026-08-06

**Authors:** Rongbo Ding, Hongyan Jia

**Affiliations:** 1 Department of Breast Surgery, First Hospital of Shanxi Medical University, Taiyuan, Shanxi, China; 2 Department of Breast Surgery, Heping Hospital Affiliated to Changzhi Medical College, Changzhi, Shanxi, China

**Keywords:** adolescent and young adult, cancer burden projection, female cancer, GLOBOCAN 2022, health disparities

## Abstract

**Objectives:**

To quantify the global, regional, and national burden of six major female cancers among adolescent and young adult (AYA) women (aged 15–39 years) in 2022, and to project incidence and mortality trends to 2050.

**Methods:**

Using GLOBOCAN 2022 estimates, we analyzed incidence, mortality, age-standardized rates, cumulative risk, and mortality-to-incidence ratios (MIRs) for breast, ovarian, corpus uteri, cervical, vaginal, and vulvar cancers across 185 countries. Burden metrics were compared across geographic regions and Human Development Index (HDI) categories. Spearman correlation analyses assessed associations between HDI and cancer burden indicators. Future cancer burden through 2050 was projected based on global demographic forecasts, assuming constant 2022 age-specific incidence and mortality rates.

**Results:**

Globally, an estimated 406,385 incident cases and 93,848 deaths from female cancers occurred among AYA women in 2022, corresponding to an ASIR of 24.8 and an ASMR of 5.7 per 100,000 population. Breast cancer accounted for the largest burden of both incident cases and deaths, whereas cervical cancer exhibited the highest mortality-to-incidence ratio, indicating disproportionately poor survival. Considerable geographic and socioeconomic disparities were observed. Very high-HDI countries had the highest incidence but the lowest mortality, whereas low-HDI countries experienced disproportionately high mortality and the highest MIR. HDI was positively associated with incidence (ASIR: ρ = 0.282; cumulative incidence: ρ = 0.302; both *P* < 0.001) but strongly inversely associated with mortality (ASMR: ρ = −0.797; cumulative mortality: ρ = −0.795) and MIR (ρ = −0.900; all *P* < 0.001). Assuming constant age-specific rates, incident cases are projected to increase by 13.7% and deaths by 27.7% globally by 2050, with the largest increases expected in Africa and low-HDI countries.

**Conclusions:**

Female cancers impose a substantial and inequitable burden among AYA women worldwide. The projected increase in burden, particularly in low-HDI countries, highlights the urgent need to strengthen equitable cancer prevention, early detection, and treatment to reduce global disparities.

## 1 Introduction

Cancer in adolescents and young adults (AYA; aged 15–39 years) is a distinct clinical entity that presents unique biological, psychosocial, and therapeutic challenges (1). AYA women are more likely than older patients to harbor germline predispositions to malignancy and experience severe treatment-related toxicities, particularly in relation to fertility and endocrine function (2, 3). As survival improves, optimizing long-term care has become critical. The reproductive consequences of cancer treatment—such as gonadotoxicity—pose significant concerns for fertility, while treatment-related side effects including fatigue, depression, and endocrine disruption can impair adherence and quality of life (4, 5). Given these complexities, understanding the epidemiology and trajectory of female-specific cancers in AYA women is essential for guiding clinical practice and improving outcomes in this vulnerable population (6).

Globally, female cancers, including breast, cervical, ovarian, uterine, vaginal, and vulvar cancers, account for 38.3% of all incident cancers in women. Breast cancer remains the most common malignancy, with nearly 2.3 million new cases in 2022, while cervical cancer remains the fourth leading cause of cancer-related death among women, particularly in low- and middle-income countries (LMICs) (7). Recent data highlight a concerning trend toward younger age at diagnosis for breast and endometrial cancers, signaling a shift in the age of onset for several malignancies (8, 9). However, these trends are not uniform, with significant disparities in incidence and outcomes, particularly driven by differences in healthcare infrastructure, early detection programs, and socio-economic factors across countries (10).

Recent analyses using socioeconomic inequality metrics have further demonstrated substantial heterogeneity in the global distribution of female cancers. Using the Relative Concentration Index, Salehi et al. reported that breast, ovarian, and uterine cancer burdens were disproportionately concentrated in higher socioeconomic settings, whereas cervical cancer remained concentrated among populations with lower socioeconomic development (11). These findings highlight the complex relationship between socioeconomic conditions and cancer epidemiology, reflecting differences in risk-factor exposure, screening availability, diagnostic capacity, and treatment accessibility (12). However, comprehensive evaluations of socioeconomic disparities across multiple female cancers among AYA women, together with projections of future burden, remain limited.

While previous analyses based on the Global Burden of Disease (GBD) study have explored trends in AYA cancer incidence and mortality, these studies rely on modeled estimates synthesized from multiple data sources and statistical frameworks. Likewise, GLOBOCAN combines the best available national cancer registry and mortality data with internationally standardized estimation methods to generate country-specific cancer burden estimates, particularly where high-quality registration data are incomplete (9, 13, 14). Consequently, differences between GBD and GLOBOCAN estimates may reflect variations in data inputs, modeling strategies, and underlying assumptions rather than differences in data quality alone. Furthermore, most previous studies have focused on the more common cancers, with limited attention to rarer but clinically important malignancies such as vaginal and vulvar cancers (15). Importantly, there remains a lack of comprehensive projections of the future burden of female cancers among AYA women, particularly analyses that disentangle the relative contributions of demographic changes and epidemiological risk factors, which are essential for informing targeted cancer control strategies and resource allocation (16).

Looking to the future, the population of women aged 15–39 years is expected to grow substantially, with the most rapid increases occurring in regions with high cancer mortality, such as sub-Saharan Africa and South Asia (17–19). Simultaneously, shifting risk factors—including rising obesity rates, delayed childbearing, and changes in reproductive behaviors—alongside expanding HPV vaccination and cervical cancer screening programs, will reshape the cancer landscape in complex ways (20–22). A rigorous projection framework is needed to understand how these demographic and epidemiological shifts will affect AYA cancer incidence and mortality, ensuring that interventions are effectively prioritized.

In this study, we used GLOBOCAN 2022 data to evaluate the global burden of six major female cancers—breast, ovarian, corpus uteri, cervical, vaginal, and vulvar—among women aged 15–39 years across 185 countries. We analyzed current cancer burden patterns and their relationship with the Human Development Index (HDI) and projected incidence and mortality to 2050. These findings aim to guide oncologists and policymakers in prioritizing interventions for AYA women worldwide, with an emphasis on ensuring equitable care and improving long-term outcomes.

## 2 Materials and methods

### 2.1 Data source

This descriptive epidemiological study utilized cancer burden estimates from GLOBOCAN 2022, developed by the International Agency for Research on Cancer (IARC) and accessed through the Global Cancer Observatory (GCO) (https://gco.iarc.fr) (7). The GLOBOCAN database provides comprehensive global estimates of cancer incidence, mortality, prevalence, and other key indicators across countries and territories worldwide (23). At the national level, cancer estimates are generated using the best available population-based data sources, including cancer registries, civil registration and vital statistics systems, and statistical modeling approaches when empirical data are incomplete or unavailable (7). These estimation procedures are adapted to the data availability and quality of individual countries or territories to ensure the most reliable assessment of cancer burden. GLOBOCAN 2022 provides estimates of cancer incidence and mortality for 185 countries and territories and 36 cancer types for the year 2022, based on the most recent available national cancer surveillance data and complementary modeling methods where necessary (14). Ethical approval and informed consent were not required for this study, as all data were obtained from publicly available sources.

### 2.2 Study population and cancer types

The present analysis focused exclusively on female cancers among women aged 15–39 years. Specifically, we examined breast cancer (International Classification of Diseases, 10th Revision [ICD-10]: C50) and five gynecological cancers: ovarian cancer (ICD-10: C56), corpus uteri cancer (ICD-10: C54), cervical cancer (ICD-10: C53), vaginal cancer (ICD-10: C52), and vulvar cancer (ICD-10: C51). These cancer types were selected based on their clinical and epidemiological relevance in young women, as well as their representation in the GLOBOCAN 2022 database (7). Incident cases and deaths for all six cancers were extracted and analyzed at the global, regional, and national levels.

### 2.3 Measures

The primary epidemiological measures extracted and analyzed included the estimated number of new cases and deaths, the age-standardized incidence rate (ASIR), the age-standardized mortality rate (ASMR), cumulative risk, and the mortality-to-incidence ratio (MIR). Age-standardized rates were calculated by adjusting crude rates using the Segi-Doll World Standard Population, enabling valid comparisons across countries and regions with differing age structures (23). Cumulative risk was defined as the probability of developing or dying from a specific cancer between ages 15 and 39 years, under the assumption that age-specific incidence or mortality rates remain constant and competing causes of death are ignored (24). The cumulative risk was calculated using the following formula:


$$Cumulative\;rate=\sum_{i}\left(r_{i}\times w_{i}\right)\;Cumulative\;risk=100\times \left(1-e^{-Cumulative\frac{rate}{100}}\right)$$

where *r_i_* is the age-specific incidence (or mortality) rate and *w_i_* is the width of each age interval. The MIR was calculated as the ratio of the ASMR to the ASIR for each cancer type, region, and HDI stratum, and serves as a proxy indicator of cancer survival and the adequacy of healthcare systems in diagnosing and treating cancer (25).

### 2.4 Geographic and human development index stratification

Cancer burden metrics were stratified by six geographic regions as defined by the United Nations: Africa, Asia, Europe, Latin America and the Caribbean, Northern America, and Oceania. Additionally, countries were stratified by their Human Development Index (HDI) level, as published by the United Nations Development Programme. HDI is a composite measure encompassing life expectancy, education, and per capita income, and countries were classified into four tiers: low HDI, medium HDI, high HDI, and very high HDI (26). HDI-stratified analyses were conducted to examine the relationship between national development status and female cancer burden across incidence, mortality, cumulative risk, and MIR.

### 2.5 Cancer burden projections to 2050

The projected incidence and mortality of female cancers in 2050 were obtained from the Cancer Tomorrow database (https://gco.iarc.fr/tomorrow/en). Future numbers of cancer cases and deaths were estimated by applying the age-specific incidence and mortality rates observed in 2022 to the projected population structure for 2050, assuming that age-specific cancer rates remain unchanged over the projection period (7, 23). For each cancer outcome, projected cases/deaths in 2050 were calculated as:


$$N_{2050}=\sum_{a}\left(r_{2022,a}\times P_{2050,\;a}\right)$$

where *N*_2050_ represents the projected number of cancer cases or deaths in 2050, *r*_2022,_*_a_* represents the age-specific incidence or mortality rate in 2022 for age group *a*, and *P*_2050,_*_a_* represents the projected population size in the corresponding age group in 2050 predicted by the United Nations Development Programme (https://hdr.undp.org/content/human-development-report-2021-22). The overall change between 2022 and 2050 was calculated as: Δ*N* = *N*_2050_ – *N*_2022_. Projections were conducted at the global, regional, Human Development Index (HDI) stratum, cancer type, and country levels.

### 2.6 Statistical analysis

To assess the relationship between HDI and country-level cancer burden metrics, Spearman rank correlation analyses were performed. Spearman correlation coefficients (ρ) and corresponding two-tailed *P* values were calculated for the following pairs: ASIR versus HDI, ASMR versus HDI, cumulative incidence versus HDI, cumulative mortality versus HDI, and MIR versus HDI. Additionally, the association between projected percentage changes in incidence and mortality and country-level HDI was evaluated using Spearman correlation. All statistical tests were two-tailed, with significance defined as *P* < 0.05. Analyses were conducted using R version 4.3.5 (R Foundation for Statistical Computing, Vienna, Austria).

## 3 Results

### 3.1 Global burden in 2022

In 2022, an estimated 406,385 incident cancer cases occurred among women aged 15–39 years globally (ASIR: 24.8 per 100,000; cumulative risk: 0.72%), resulting in 93,848 deaths (ASMR: 5.7 per 100,000; cumulative mortality risk: 0.17%). The overall MIR was 0.23 (**Table 1**).

**Table 1:** Estimated number of cases, ASIR, ASMR, cumulative risk, and mortality-to-incidence ratio for female cancer among women aged 15-39 years old in 2022.

Regions	Incidence			Mortality			Mortality-to-incidence ratios
	Number of Cases	ASIR per 100,000	Cumulative risk	Number of Cases	ASMR per 100,000	Cumulative risk	
World	406385	24.8	0.72	93848	5.7	0.17	0.23
Region							
Africa	72660	27.0	0.78	28885	10.7	0.31	0.4
Asia	206194	20.7	0.60	46313	4.6	0.14	0.22
Europe	52465	36.6	1.1	6286	4.2	0.12	0.11
Latin America and the Caribbean	45931	31.2	0.89	9423	6.3	0.18	0.2
Northern America	25880	35.0	1.0	2331	3.1	0.09	0.09
Oceania	3255	36.6	1.1	610	6.9	0.20	0.19
Human development index (HDI)							
Very high HDI countries	111309	34.9	1.0	12815	3.9	0.12	0.11
High HDI countries	145880	24.6	0.71	27345	4.5	0.13	0.18
Medium HDI countries	94425	19.0	0.55	30817	6.2	0.18	0.33
Low HDI countries	54611	24.1	0.69	22830	10.1	0.29	0.42
Cancer type							
Breast	245827	14.9	0.43	48658	2.9	0.09	0.19
Ovary	37703	2.4	0.07	9846	0.63	0.02	0.26
Corpus uteri	13142	0.80	0.02	1571	0.10	0.00	0.13
Cervix uteri	105728	6.5	0.19	32575	2.0	0.06	0.31
Vagina	1171	0.07	0.00	366	0.02	0.00	0.29
Vulva	2814	0.17	0.00	832	0.05	0.00	0.29

ASIR, Age-standardized incidence rate; ASMR, Age-standardized mortality rate; HDI, Human development index.

Breast cancer accounted for the greatest share of the burden, with 245,827 cases (ASIR: 14.9 per 100,000) and 48,658 deaths (ASMR: 2.9 per 100,000). Cervical cancer ranked second in incidence (105,728 cases; ASIR: 6.5 per 100,000) and deaths (32,575 cases; ASIR: 2.0 per 100,000), but had the highest MIR (0.31), reflecting higher mortality relative to incidence. Ovarian cancer contributed 37,703 cases and 9,846 deaths (MIR: 0.26), while corpus uteri cancer was less common (13,142 cases; MIR: 0.13). Vaginal and vulvar cancers were rare but notable for their comparatively high MIRs (both 0.29).

### 3.2 Geographical variation in 2022

The global distribution of cancer burden varied considerably by geographical region (**Table 1**; **Supplementary Table 1**). Incidence was highest in Europe and Oceania (ASIR: 36.6 per 100,000) and Northern America (ASIR: 35.0 per 100,000), and lowest in Asia (ASIR: 20.7 per 100,000; **Table 1**). Mortality, however, followed a markedly different pattern: Africa recorded the highest ASMR (10.7 per 100,000) and MIR (0.40), while Northern America had the lowest ASMR (3.1 per 100,000) and MIR (0.09).

**Figure 1:**
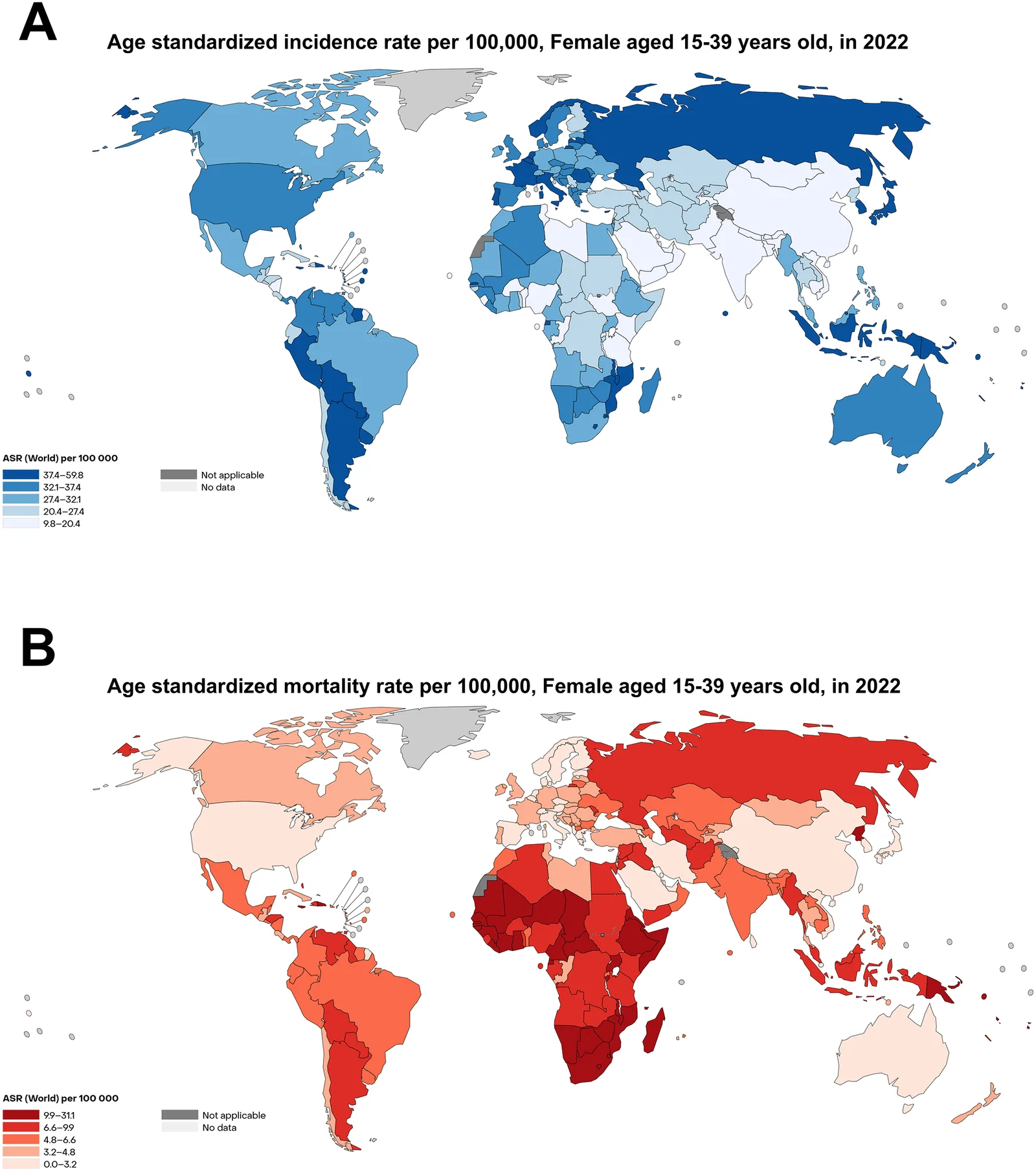
Global distribution of age-standardized incidence and mortality rates of female cancers among women aged 15–39 years in 2022. **(A)** Age-standardized incidence rates (per 100,000 population) and **(B)** age-standardized mortality rates (per 100,000 population), both standardized to the world standard population. Data are presented at the country level and categorized into ranges as indicated in the legends; areas with no available data or not applicable are shown separately. Data were obtained from the GLOBOCAN 2022 database, compiled by the International Agency for Research on Cancer (IARC) and accessed via the Global Cancer Observatory.

At the country level, the highest ASIRs were observed in Fiji (59.8 per 100,000) and Malawi (59.0 per 100,000), while Sri Lanka recorded the lowest (9.8 per 100,000; **Figure 1**; **Supplementary Table 1**). Mortality was most severe in sub-Saharan Africa, with Malawi (31.1 per 100,000) and Mozambique (25.9 per 100,000) reporting the highest ASMRs. MIRs were highest in Chad (0.63) and the Central African Republic (0.54), and lowest in Norway and Cyprus (both 0.04).

### 3.3 Burden by development level in 2022

**Figure 2:**
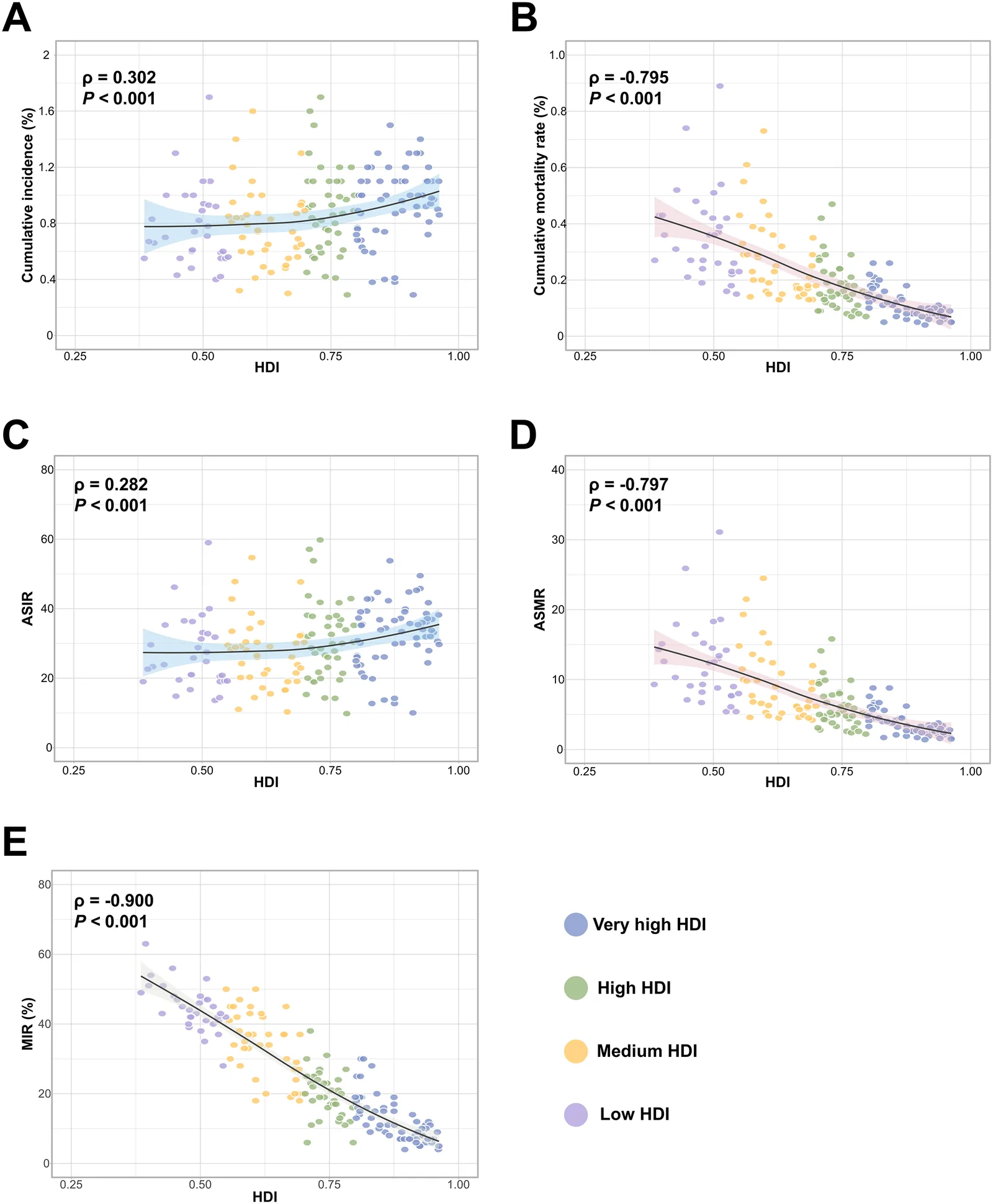
Association between female cancer burden and the Human Development Index (HDI) among women aged 15–39 years in 2022. **(A)** Relationship between cumulative incidence (%) and HDI. **(B)** Relationship between cumulative mortality (%) and HDI. **(C)** Relationship between age-standardized incidence rate (ASIR) and HDI. **(D)** Relationship between age-standardized mortality rate (ASMR) and HDI. **(E)** Relationship between mortality-to-incidence ratio (MIR, %) and HDI. Each point represents a country, stratified by HDI categories (low, medium, high, and very high). Spearman correlation coefficients (ρ) and corresponding P values are shown in each panel, indicating positive correlations for incidence measures and inverse correlations for mortality and MIR with increasing HDI.

Very high HDI countries had the highest incidence (ASIR: 34.9 per 100,000) but the lowest mortality (ASMR: 3.9 per 100,000; MIR: 0.11; **Table 1**). By contrast, low HDI countries, despite having a moderate incidence (ASIR: 24.1 per 100,000), bore a disproportionately high mortality burden (MIR: 0.42). Medium HDI countries showed the lowest incidence (ASIR: 19.0 per 100,000) alongside elevated mortality (ASMR: 6.2 per 100,000; MIR: 0.33). These patterns were confirmed by correlation analyses (**Figure 2**). Cumulative incidence (ρ = 0.302, *P* < 0.001) and ASIR (ρ = 0.282, *P<* 0.001) were positively correlated with HDI (**Figures 2A, C**). Conversely, cumulative mortality (ρ = −0.795, P< 0.001), ASMR (ρ = −0.797, *P<* 0.001), and MIR (ρ = −0.900, *P* < 0.001) showed strong inverse associations with HDI (**Figures 2B, D, E**).

### 3.4 Global projections to 2050

#### 3.4.1 Overall trend

Assuming constant age-specific incidence and mortality rates, the global burden of female cancers among women aged 15–39 years is projected to increase substantially by 2050 **Table 2**). Incident cases are expected to reach 462,151, representing an increase of 55,766 cases (13.7%), while cancer deaths are projected to increase to 119,845, corresponding to 25,997 additional deaths (27.7%).

**Table 2:** Projected number of female cancer cases in women aged 15–39 years in 2050 and percentage change from 2022.

Regions	Incidence		Mortality	
	Number of Case in 2050	Change in number of cases due to demographic changes between 2022 and 2050 (Percentage change)	Number of Case in 2050	Change in number of deaths due to demographic changes between 2022 and 2050 (Percentage change)
World	462151	55766 (13.72%)	119845	25997 (27.7%)
Region				
Africa	142423	69763 (96.01%)	56713	27828 (96.34%)
Asia	204022	-2172 (-1.05%)	46006	-307 (-0.66%)
Europe	42498	-9967 (-19%)	5074	-1212 (-19.28%)
Latin America and the Caribbean	43021	-2910 (-6.34%)	8930	-493 (-5.23%)
Northern America	26146	266 (1.03%)	2366	35 (1.5%)
Oceania	4041	786 (24.15%)	756	146 (23.93%)
Human development index (HDI)				
Very high HDI countries	100152	-11157 (-10.02%)	11564	-1251 (-9.76%)
High HDI countries	126723	-19157 (-13.13%)	23843	-3502 (-12.81%)
Medium HDI countries	109573	15148 (16.04%)	35906	5089 (16.51%)
Low HDI countries	113644	59033 (108.1%)	47696	24866 (108.92%)
Cancer type				
Breast	278814	32987 (13.42%)	63841	15183 (31.2%)
Ovary	41113	3410 (9.04%)	11246	1400 (14.22%)
Corpus uteri	13535	393 (0.03%)	1599	28 (1.78%)
Cervix uteri	123133	17405 (16.46%)	41338	8763 (26.9%)
Vagina	1458	287 (24.51%)	477	111 (30.33%)
Vulva	4043	1229 (43.67%)	1344	512 (61.54%)

HDI, Human development index.

#### 3.4.2 Regional and development-level projections

Marked geographic heterogeneity was observed. Africa is projected to experience the greatest increase in cancer burden, with incident cases and deaths nearly doubling compared with 2022 (+96.0% and +96.3%, respectively), whereas Europe is expected to show the largest decline (−19.0% for incidence and −19.3% for mortality). Asia, Latin America and the Caribbean, and North America are projected to experience relatively small changes, while Oceania is expected to show moderate increases **Table 2**). Substantial disparities were also evident across HDI categories. Low-HDI countries are projected to experience the largest increases, with incident cases and deaths increasing by 108.1% and 108.9%, respectively. In contrast, very high- and high-HDI countries are projected to experience declines in both incidence (−10.0% and −13.1%) and mortality (−9.8% and −12.8%), whereas medium-HDI countries are expected to show modest increases of approximately 16% (**Table 2**).

#### 3.4.3 Country-level projections

At the country level, substantial heterogeneity in projected demographic changes was observed (**Supplementary Table 2**). The largest projected increases in both incident cases and deaths were concentrated in sub-Saharan Africa, including Niger, Somalia, Mali, the Democratic Republic of the Congo, Chad, Angola, Mozambique, Uganda, Nigeria, and Tanzania, where projected increases frequently exceeded 100%. By contrast, many countries in Europe and East Asia, including Serbia, the Republic of Moldova, Ukraine, the Republic of Korea, Japan, China, Italy, and Poland, are projected to experience declining numbers of cases and deaths.

**Figure 3:**
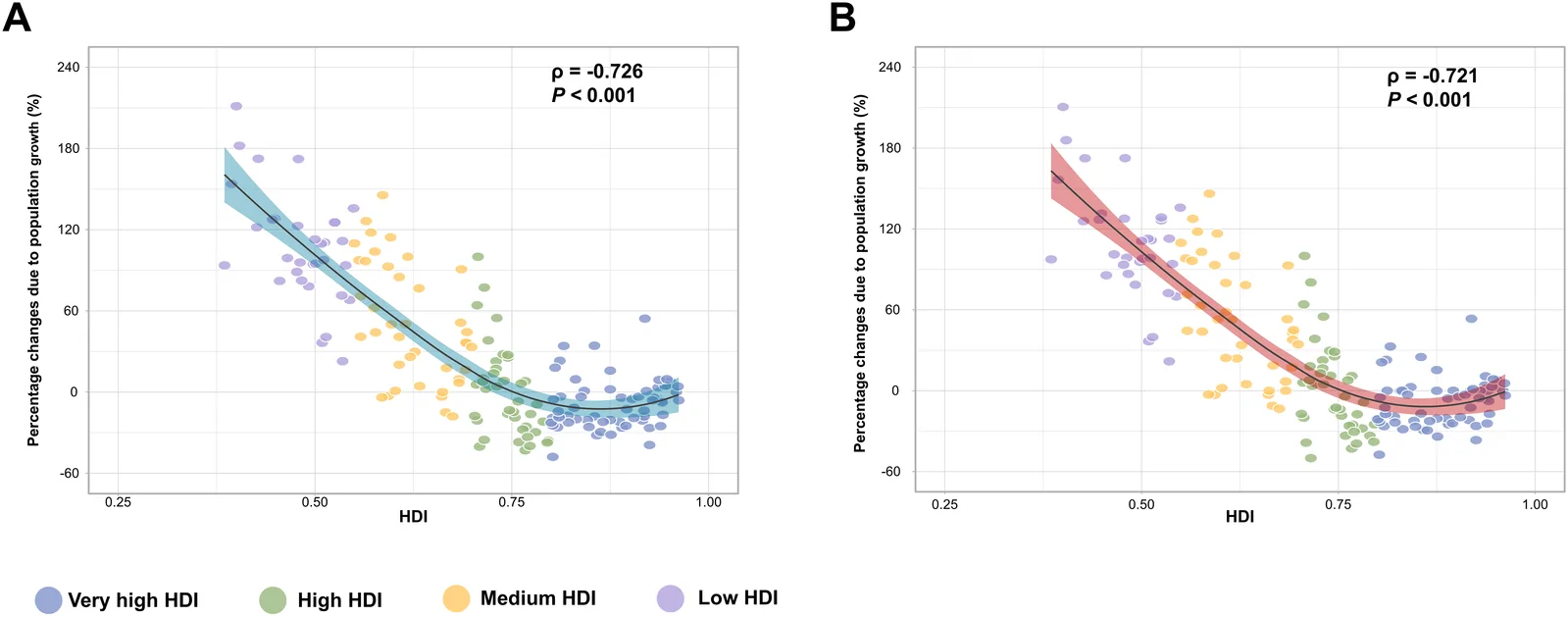
Association of HDI with projected percentage changes in female cancer burden among women aged 15–39 years, 2022–2050. **(A)** Association between HDI and the percentage change in incident cancer cases attributable to demographic change. **(B)** Association between HDI and the percentage change in cancer deaths attributable to demographic change. Spearman correlation coefficients (ρ) and corresponding P values are shown in each panel.

Consistent with these geographic patterns, projected demographic changes were strongly and inversely associated with HDI (**Figure 3**). Higher HDI was associated with smaller projected increases—or even declines—in both cancer incidence (Spearman ρ = −0.726, *P* < 0.001) and mortality (Spearman ρ = −0.721, *P* < 0.001), indicating that future increases in the burden of female cancers among AYA women are expected to be concentrated predominantly in lower-HDI settings.

#### 3.4.4 Projections by cancer type

Breast cancer is projected to remain the leading contributor to the future cancer burden, accounting for 278,814 incident cases and 63,841 deaths by 2050. Cervical cancer is projected to rank second, with 123,133 incident cases and 41,338 deaths, followed by ovarian cancer (41,113 cases and 11,246 deaths). Although less common, vulvar and vaginal cancers are projected to exhibit the largest relative increases, particularly for mortality (61.5% and 30.3%, respectively) (**Table 2**).

Patterns varied considerably by cancer type across HDI levels (**Supplementary Table 3**). For breast cancer, incident cases are projected to increase by 108.2% in low-HDI countries but decline by approximately 10–13% in very high- and high-HDI countries. Similar trends were observed for ovarian and cervical cancers, with low-HDI countries experiencing increases exceeding 100% for both incidence and mortality, whereas very high- and high-HDI countries are projected to experience consistent declines. Medium-HDI countries are expected to show moderate increases of approximately 14–17% across all three cancers.

## 4 Discussion

This study provides an updated global assessment of six major female cancers among AYA women, revealing substantial disparities between cancer occurrence and outcomes across regions. Although breast cancer accounted for most incident cases (60.5%) and deaths (51.8%), cervical cancer showed a disproportionately high mortality burden relative to incidence, reflected by its higher MIR (0.31 vs 0.19 for breast cancer). A strong socioeconomic gradient was evident: very high-HDI countries had the highest incidence but the lowest MIR (0.11), whereas low-HDI countries experienced substantially poorer population-level outcomes (MIR, 0.42). By 2050, incident cases and deaths are projected to rise 13.72% and 27.7%, driven by demographic effect in low HDI regions. These differences may partly reflect variation in early detection, access to treatment, healthcare infrastructure, and data completeness. Collectively, these findings underscore the growing need for equitable cancer prevention and healthcare investment in resource-limited settings.

Our findings are consistent with previous studies demonstrating substantial socioeconomic inequalities in the global distribution of female cancers (11). Breast, ovarian, and corpus uteri cancers are more frequently diagnosed in countries with higher socioeconomic development, likely reflecting differences in reproductive patterns, obesity prevalence, diagnostic intensity, and cancer registration systems (27–31). In contrast, cervical cancer remains disproportionately concentrated in less-developed settings because of persistent inequities in HPV vaccination, cervical screening, and timely treatment (32). Extending these observations, our study identified a clear incidence–mortality paradox among AYA women: countries with very high HDI exhibited higher incidence but markedly lower mortality-to-incidence ratios, while low-HDI countries experienced disproportionate mortality burdens. These findings suggest that differences in early detection, diagnostic capacity, treatment accessibility, and overall health-system performance continue to shape global inequalities in cancer outcomes among AYA women (23, 33).

The predominance of breast cancer among AYA women mirrors findings from population-based registries in North America and Europe, where it has surpassed leukemia as the most common malignancy in women under 40 (34, 35). The trend toward younger-onset breast cancer observed here aligns with recent analyses from large national registries in the United States (36), South Korea (37), and the France (38), which report rising incidence in women under 40, and with modeled estimates from the GBD study (39). Moreover, breast cancer is projected to remain the leading contributor to the future cancer burden through 2050. Although our projections assume constant age-specific incidence and mortality rates, the increasing number of cases and deaths reflects the anticipated growth of the global AYA female population, particularly in low- and middle-income countries. These findings emphasize the importance of strengthening awareness, timely diagnosis, genetic risk assessment for high-risk individuals, and equitable access to multidisciplinary treatment for young women with breast cancer.

Cervical cancer represents a contrasting pattern. Although it ranked second in both incidence and mortality, it exhibited the highest mortality-to-incidence ratio among the major female cancers, indicating substantially poorer survival than breast cancer. This disparity likely reflects delayed diagnosis, limited screening coverage, inadequate HPV vaccination, and restricted access to effective treatment in resource-limited settings (32). While high- and very high-HDI countries are expected to experience stable or declining cervical cancer burden because of successful implementation of HPV vaccination and organized screening programs (22). low-HDI countries are projected to experience substantial increases in both incident cases and deaths. These findings reinforce the importance of accelerating the WHO strategy for cervical cancer elimination by expanding HPV vaccination, improving screening coverage, and ensuring timely treatment of precancerous lesions and invasive disease, particularly in sub-Saharan Africa and other underserved regions (40).

Although vaginal and vulvar cancers contributed relatively few cases, they demonstrated comparatively high mortality-to-incidence ratios and are projected to increase in absolute number, particularly in lower-HDI countries. Given the rarity of these malignancies, limited awareness, delayed diagnosis, and restricted specialist care may contribute disproportionately to poor outcomes. Strengthening gynecologic oncology services and improving access to specialized care in resource-constrained settings may therefore yield meaningful improvements in survival (41–43).

This study has several notable strengths. First, the GLOBOCAN 2022 combines the best available population-based cancer registry data with standardized statistical estimation methods to generate comprehensive cancer burden estimates across 185 countries. Although estimates for some countries rely on modeling because of incomplete cancer registration, GLOBOCAN provides a consistent and internationally recognized framework for global cancer surveillance and comparison. Second, by analyzing all six major female cancers simultaneously, including the often-overlooked vaginal and vulvar malignancies, the study presents a comprehensive overview of the adolescent and young adult (AYA) female cancer landscape that previous single-cancer analyses could not capture. However, several limitations warrant consideration. First, although GLOBOCAN 2022 integrates the best available population-based cancer registry and vital registration data compiled by the IARC, estimates for countries with incomplete cancer surveillance rely on statistical modeling approaches, including mortality-to-incidence ratio methods, extrapolation, and borrowing information from comparable populations. Consequently, the projected estimates in this study inherit uncertainty from the underlying GLOBOCAN estimates, and the magnitude of this uncertainty is not uniform across countries. It is likely greatest in regions with limited cancer registration infrastructure—particularly sub-Saharan Africa and parts of South Asia—which also account for the largest projected increases in cancer burden. Therefore, projections for these settings should be interpreted as indicative of the direction and relative magnitude of future burden rather than precise quantitative forecasts. However, uncertainty intervals could not be calculated for the present analyses because their estimation requires corrections for registry coverage, reporting delay, and data quality, which have been evaluated only for the overall population and are not available for the adolescent and young adult population. Second, our projections assume age-specific cancer rates remain at 2022 levels and do not account for future changes in risk factors, healthcare capacity, policy interventions, or technological innovations, making them a reference scenario rather than true forecasts. Moreover, our projections were generated using the IARC decomposition framework, which provides deterministic reference scenario estimates but does not quantify statistical uncertainty. Therefore, only point estimates are presented and should be interpreted cautiously, particularly for countries with limited cancer registry coverage. Third, the HDI may obscure within-country disparities; high-HDI nations can still harbor underserved populations with cancer outcomes comparable to low-HDI countries, and sub-national granularity was not available. Finally, although MIR is commonly used as a population-level proxy of cancer outcomes in global epidemiological studies, it should not be regarded as a direct measure of survival or case fatality. MIR is influenced by multiple factors, including cancer registration completeness, diagnostic practices, mortality certification, stage distribution, treatment availability, and modeling approaches used in data-limited settings. Therefore, MIR should be interpreted cautiously as an ecological indicator of cancer outcomes rather than an individual-level measure of prognosis, particularly in regions with incomplete mortality surveillance.

## 5 Conclusions

In conclusion, our findings demonstrate substantial and persistent global inequalities in female cancers among AYA women. While breast cancer remains the dominant contributor to both incidence and mortality, cervical cancer continues to impose a disproportionate mortality burden in socioeconomically disadvantaged settings. As the global burden shifts increasingly toward low-HDI countries over the coming decades, expanding equitable access to prevention, early diagnosis, and effective cancer treatment will be essential to reducing disparities and improving outcomes for young women worldwide.

## Data Availability

The original contributions presented in the study are included in the article/**Supplementary Material**. Further inquiries can be directed to the corresponding authors.
