# Supplementary material for: Female cancers in adolescent and young adult women: global inequities and 2050 burden estimates from GLOBOCAN 2022
Source: Front Oncol. 2026 Aug 6;16:1896950. doi: 10.3389/fonc.2026.1896950 (PMC13489919; doi:10.3389/fonc.2026.1896950)
Supplement: Supplementary file 2 [file SupplementaryFile1.docx]

**Supplementary Table S1. Estimated number of cases, ASIR, ASMR, cumulative risk, and mortality-to-incidence ratio for female cancer among women aged 15-39 years old in 2022, by country**

|  | Incidence | | |  | Mortality | | |  | Mortality-to-incidence ratios |
| --- | --- | --- | --- | --- | --- | --- | --- | --- | --- |
|  | Number of Cases | ASIR per 100,000 | Cumulative risk |  | Number of Cases | ASMR per 100,000 | Cumulative risk |  |  |
| Afghanistan | 1521 | 20.9 | 0.60 |  | 586 | 8.2 | 0.24 |  | 0.39 |
| Albania | 192 | 35.2 | 1.0 |  | 12 | 2.2 | 0.07 |  | 0.06 |
| Algeria | 3622 | 34.9 | 1.0 |  | 858 | 8.2 | 0.24 |  | 0.23 |
| Angola | 1767 | 28.1 | 0.79 |  | 612 | 9.8 | 0.28 |  | 0.35 |
| Argentina | 4225 | 44.7 | 1.3 |  | 851 | 8.8 | 0.26 |  | 0.2 |
| Armenia | 103 | 14.2 | 0.41 |  | 21 | 2.6 | 0.08 |  | 0.18 |
| Australia | 1819 | 33.1 | 0.97 |  | 136 | 2.4 | 0.07 |  | 0.07 |
| Austria | 558 | 31.7 | 0.92 |  | 46 | 2.5 | 0.07 |  | 0.08 |
| Azerbaijan | 586 | 23.1 | 0.66 |  | 138 | 5.3 | 0.15 |  | 0.23 |
| Bahamas | 31 | 35.5 | 1.0 |  | 6 | 6.7 | 0.21 |  | 0.19 |
| Bahrain | 48 | 12.7 | 0.38 |  | 7 | 1.9 | 0.06 |  | 0.15 |
| Bangladesh | 6619 | 16.5 | 0.48 |  | 2456 | 6.1 | 0.18 |  | 0.37 |
| Barbados | 23 | 42.9 | 1.2 |  | 3 | 5.2 | 0.16 |  | 0.12 |
| Belarus | 627 | 30.1 | 0.87 |  | 95 | 4.3 | 0.13 |  | 0.14 |
| Belgium | 838 | 38.6 | 1.1 |  | 49 | 2.2 | 0.07 |  | 0.06 |
| Belize | 21 | 21.5 | 0.63 |  | 6 | 5.9 | 0.17 |  | 0.27 |
| Benin | 316 | 13.6 | 0.40 |  | 125 | 5.4 | 0.16 |  | 0.4 |
| Bhutan | 20 | 10.3 | 0.30 |  | 9 | 4.6 | 0.14 |  | 0.45 |
| Bolivia (Plurinational State of) | 1200 | 47.7 | 1.3 |  | 245 | 9.6 | 0.29 |  | 0.2 |
| Bosnia Herzegovina | 206 | 33.9 | 0.97 |  | 25 | 3.8 | 0.11 |  | 0.11 |
| Botswana | 187 | 32.5 | 0.95 |  | 69 | 12.0 | 0.35 |  | 0.37 |
| Brazil | 14734 | 29.9 | 0.86 |  | 3239 | 6.4 | 0.19 |  | 0.21 |
| Brunei Darussalam | 43 | 42.8 | 1.2 |  | 4 | 4.0 | 0.11 |  | 0.09 |
| Bulgaria | 384 | 30.4 | 0.89 |  | 66 | 5.0 | 0.15 |  | 0.16 |
| Burkina Faso | 579 | 14.8 | 0.43 |  | 274 | 7.1 | 0.21 |  | 0.48 |
| Burundi | 597 | 23.9 | 0.70 |  | 256 | 10.3 | 0.31 |  | 0.43 |
| Cambodia | 898 | 20.3 | 0.59 |  | 300 | 6.6 | 0.20 |  | 0.33 |
| Cameroon | 1576 | 29.2 | 0.84 |  | 735 | 13.6 | 0.39 |  | 0.47 |
| Canada | 2471 | 31.9 | 0.92 |  | 277 | 3.5 | 0.10 |  | 0.11 |
| Cape Verde | 24 | 16.6 | 0.50 |  | 8 | 6.2 | 0.17 |  | 0.37 |
| Central African Republic | 200 | 23.3 | 0.66 |  | 106 | 12.6 | 0.36 |  | 0.54 |
| Chad | 651 | 22.6 | 0.67 |  | 408 | 14.3 | 0.43 |  | 0.63 |
| Chile | 1086 | 26.1 | 0.74 |  | 177 | 4.2 | 0.12 |  | 0.16 |
| China | 57052 | 19.5 | 0.56 |  | 7084 | 2.4 | 0.07 |  | 0.12 |
| Colombia | 3794 | 32.6 | 0.93 |  | 648 | 5.5 | 0.16 |  | 0.17 |
| Comoros | 61 | 33.5 | 0.95 |  | 18 | 10.0 | 0.29 |  | 0.3 |
| Congo, Democratic Republic of | 3369 | 21.0 | 0.61 |  | 1477 | 9.3 | 0.27 |  | 0.44 |
| Congo, Republic of | 117 | 11.0 | 0.32 |  | 50 | 4.6 | 0.14 |  | 0.42 |
| Costa Rica | 283 | 23.8 | 0.68 |  | 55 | 4.5 | 0.13 |  | 0.19 |
| Côte d'Ivoire | 1533 | 29.5 | 0.85 |  | 769 | 14.8 | 0.43 |  | 0.5 |
| Croatia | 254 | 34.4 | 1.0 |  | 24 | 3.2 | 0.09 |  | 0.09 |
| Cuba | 540 | 25.8 | 0.73 |  | 96 | 4.4 | 0.13 |  | 0.17 |
| Cyprus | 111 | 39.9 | 1.2 |  | 5 | 1.7 | 0.05 |  | 0.04 |
| Czechia | 642 | 33.4 | 0.96 |  | 52 | 2.5 | 0.08 |  | 0.07 |
| Denmark | 374 | 36.9 | 1.1 |  | 21 | 2.1 | 0.06 |  | 0.06 |
| Djibouti | 63 | 27.5 | 0.78 |  | 30 | 13.0 | 0.37 |  | 0.47 |
| Dominican Republic | 983 | 41.7 | 1.2 |  | 238 | 9.9 | 0.29 |  | 0.24 |
| Ecuador | 1041 | 26.6 | 0.76 |  | 199 | 5.0 | 0.15 |  | 0.19 |
| Egypt | 6418 | 28.5 | 0.82 |  | 1750 | 7.8 | 0.22 |  | 0.27 |
| El Salvador | 396 | 26.1 | 0.74 |  | 75 | 4.9 | 0.15 |  | 0.19 |
| Equatorial Guinea | 111 | 38.7 | 1.1 |  | 48 | 16.7 | 0.48 |  | 0.43 |
| Eritrea | 206 | 28.8 | 0.82 |  | 89 | 12.4 | 0.36 |  | 0.43 |
| Estonia | 80 | 30.1 | 0.89 |  | 8 | 2.8 | 0.09 |  | 0.09 |
| Eswatini | 146 | 54.7 | 1.6 |  | 66 | 24.5 | 0.73 |  | 0.45 |
| Ethiopia | 7341 | 31.3 | 0.89 |  | 3384 | 14.5 | 0.42 |  | 0.46 |
| Fiji | 114 | 59.8 | 1.7 |  | 31 | 15.8 | 0.47 |  | 0.26 |
| Finland | 248 | 24.4 | 0.72 |  | 17 | 1.6 | 0.05 |  | 0.07 |
| France (metropolitan) | 5250 | 44.8 | 1.3 |  | 398 | 3.3 | 0.10 |  | 0.07 |
| France, Guadeloupe | 21 | 37.1 | 1.1 |  | 4 | 6.8 | 0.21 |  | 0.18 |
| France, La Réunion | 44 | 27.4 | 0.81 |  | 7 | 4.2 | 0.13 |  | 0.15 |
| France, Martinique | 19 | 33.8 | 1.0 |  | 3 | 5.2 | 0.16 |  | 0.15 |
| French Guyana | 12 | 19.0 | 0.57 |  | 1 | 1.6 | 0.05 |  | 0.08 |
| French Polynesia | 18 | 27.5 | 0.83 |  | 3 | 4.4 | 0.14 |  | 0.16 |
| Gabon | 162 | 32.0 | 0.91 |  | 50 | 9.7 | 0.28 |  | 0.3 |
| Gaza Strip and West Bank | 207 | 19.2 | 0.56 |  | 51 | 4.8 | 0.14 |  | 0.25 |
| Georgia | 185 | 23.6 | 0.68 |  | 34 | 4.1 | 0.12 |  | 0.17 |
| Germany | 4175 | 27.9 | 0.81 |  | 515 | 3.4 | 0.10 |  | 0.12 |
| Ghana | 1870 | 29.1 | 0.83 |  | 702 | 10.9 | 0.32 |  | 0.37 |
| Greece | 640 | 37.2 | 1.1 |  | 49 | 2.6 | 0.08 |  | 0.07 |
| Guam | 11 | 31.7 | 0.97 |  | 0 | 0.00 | 0.00 |  | 0 |
| Guatemala | 920 | 22.4 | 0.65 |  | 183 | 4.5 | 0.13 |  | 0.2 |
| Guinea | 949 | 36.5 | 1.0 |  | 426 | 16.5 | 0.48 |  | 0.45 |
| Guinea-Bissau | 108 | 25.8 | 0.74 |  | 45 | 10.8 | 0.32 |  | 0.42 |
| Guyana | 49 | 32.7 | 0.93 |  | 11 | 7.6 | 0.23 |  | 0.23 |
| Haiti | 547 | 20.5 | 0.59 |  | 202 | 7.6 | 0.22 |  | 0.37 |
| Honduras | 486 | 21.2 | 0.61 |  | 205 | 9.0 | 0.25 |  | 0.42 |
| Hungary | 630 | 36.8 | 1.1 |  | 67 | 3.7 | 0.11 |  | 0.1 |
| Iceland | 21 | 30.6 | 0.90 |  | 2 | 2.8 | 0.09 |  | 0.09 |
| India | 47968 | 15.5 | 0.45 |  | 15954 | 5.2 | 0.15 |  | 0.34 |
| Indonesia | 22604 | 38.1 | 1.1 |  | 5644 | 9.4 | 0.27 |  | 0.25 |
| Iran, Islamic Republic of | 4795 | 21.4 | 0.61 |  | 774 | 3.1 | 0.09 |  | 0.14 |
| Iraq | 2013 | 23.9 | 0.69 |  | 591 | 7.0 | 0.21 |  | 0.29 |
| Ireland | 314 | 32.0 | 0.94 |  | 35 | 3.4 | 0.10 |  | 0.11 |
| Israel | 488 | 29.6 | 0.86 |  | 58 | 3.5 | 0.10 |  | 0.12 |
| Italy | 3807 | 39.1 | 1.1 |  | 278 | 2.7 | 0.08 |  | 0.07 |
| Jamaica | 378 | 57.1 | 1.6 |  | 96 | 14.1 | 0.42 |  | 0.25 |
| Japan | 9005 | 45.8 | 1.3 |  | 516 | 2.5 | 0.08 |  | 0.05 |
| Jordan | 604 | 27.2 | 0.79 |  | 148 | 6.7 | 0.19 |  | 0.25 |
| Kazakhstan | 953 | 21.5 | 0.63 |  | 284 | 6.4 | 0.19 |  | 0.3 |
| Kenya | 2384 | 20.2 | 0.60 |  | 911 | 7.7 | 0.23 |  | 0.38 |
| Korea, Democratic People's Republic of | 1268 | 23.6 | 0.68 |  | 599 | 11.2 | 0.32 |  | 0.47 |
| Korea, Republic of | 4689 | 49.5 | 1.4 |  | 251 | 2.6 | 0.08 |  | 0.05 |
| Kuwait | 106 | 12.7 | 0.38 |  | 15 | 1.7 | 0.05 |  | 0.13 |
| Kyrgyzstan | 348 | 22.9 | 0.65 |  | 66 | 4.2 | 0.13 |  | 0.18 |
| Lao People's Democratic Republic | 437 | 26.0 | 0.74 |  | 107 | 6.3 | 0.19 |  | 0.24 |
| Latvia | 146 | 42.3 | 1.2 |  | 17 | 4.5 | 0.14 |  | 0.11 |
| Lebanon | 320 | 20.0 | 0.60 |  | 80 | 5.0 | 0.15 |  | 0.25 |
| Lesotho | 189 | 40.0 | 1.1 |  | 88 | 18.6 | 0.54 |  | 0.47 |
| Liberia | 360 | 36.3 | 1.0 |  | 150 | 15.2 | 0.44 |  | 0.42 |
| Libya | 315 | 18.9 | 0.55 |  | 71 | 4.2 | 0.12 |  | 0.22 |
| Lithuania | 170 | 36.6 | 1.1 |  | 29 | 6.2 | 0.18 |  | 0.17 |
| Luxembourg | 49 | 34.0 | 1.0 |  | 4 | 2.6 | 0.08 |  | 0.08 |
| Madagascar | 1841 | 33.1 | 0.94 |  | 690 | 12.5 | 0.36 |  | 0.38 |
| Malawi | 2300 | 59.0 | 1.7 |  | 1209 | 31.1 | 0.89 |  | 0.53 |
| Malaysia | 2475 | 30.8 | 0.89 |  | 610 | 7.6 | 0.22 |  | 0.25 |
| Maldives | 45 | 37.5 | 1.1 |  | 8 | 6.1 | 0.19 |  | 0.16 |
| Mali | 1246 | 35.3 | 1.0 |  | 632 | 17.9 | 0.52 |  | 0.51 |
| Malta | 39 | 45.3 | 1.3 |  | 3 | 3.1 | 0.10 |  | 0.07 |
| Mauritania | 273 | 28.3 | 0.81 |  | 111 | 11.5 | 0.33 |  | 0.41 |
| Mauritius | 69 | 26.5 | 0.77 |  | 13 | 4.9 | 0.15 |  | 0.18 |
| Mexico | 8112 | 27.8 | 0.80 |  | 1435 | 4.8 | 0.14 |  | 0.17 |
| Mongolia | 118 | 14.3 | 0.42 |  | 27 | 3.3 | 0.10 |  | 0.23 |
| Montenegro | 25 | 19.8 | 0.59 |  | 7 | 5.6 | 0.16 |  | 0.28 |
| Morocco | 2534 | 30.1 | 0.87 |  | 516 | 6.1 | 0.18 |  | 0.2 |
| Mozambique | 2804 | 46.2 | 1.3 |  | 1578 | 25.9 | 0.74 |  | 0.56 |
| Myanmar | 3709 | 30.4 | 0.86 |  | 1218 | 9.9 | 0.29 |  | 0.33 |
| Namibia | 209 | 36.0 | 1.0 |  | 72 | 12.4 | 0.36 |  | 0.34 |
| Nepal | 1106 | 14.4 | 0.41 |  | 409 | 5.3 | 0.16 |  | 0.37 |
| New Caledonia | 25 | 41.4 | 1.2 |  | 5 | 7.9 | 0.24 |  | 0.19 |
| New Zealand | 344 | 36.2 | 1.1 |  | 37 | 3.8 | 0.11 |  | 0.1 |
| Nicaragua | 302 | 19.0 | 0.55 |  | 91 | 5.7 | 0.17 |  | 0.3 |
| Niger | 1190 | 29.6 | 0.83 |  | 608 | 15.1 | 0.43 |  | 0.51 |
| Nigeria | 7187 | 19.0 | 0.55 |  | 2887 | 7.7 | 0.23 |  | 0.41 |
| North Macedonia | 166 | 36.8 | 1.1 |  | 23 | 4.8 | 0.15 |  | 0.13 |
| Norway | 405 | 38.2 | 1.1 |  | 18 | 1.7 | 0.05 |  | 0.04 |
| Oman | 214 | 20.3 | 0.60 |  | 64 | 6.0 | 0.18 |  | 0.3 |
| Pakistan | 9112 | 19.2 | 0.55 |  | 2533 | 5.4 | 0.15 |  | 0.28 |
| Panama | 337 | 37.3 | 1.0 |  | 57 | 6.1 | 0.18 |  | 0.16 |
| Papua New Guinea | 793 | 42.8 | 1.2 |  | 357 | 19.3 | 0.55 |  | 0.45 |
| Paraguay | 887 | 53.8 | 1.5 |  | 166 | 9.9 | 0.29 |  | 0.18 |
| Peru | 2845 | 38.0 | 1.1 |  | 478 | 6.3 | 0.18 |  | 0.17 |
| Philippines | 7269 | 30.3 | 0.89 |  | 1480 | 6.2 | 0.18 |  | 0.2 |
| Poland | 2343 | 29.9 | 0.87 |  | 285 | 3.4 | 0.10 |  | 0.11 |
| Portugal | 906 | 53.8 | 1.5 |  | 80 | 4.5 | 0.13 |  | 0.08 |
| Puerto Rico | 147 | 31.1 | 0.91 |  | 23 | 4.8 | 0.14 |  | 0.15 |
| Qatar | 70 | 13.7 | 0.40 |  | 12 | 2.1 | 0.07 |  | 0.15 |
| Republic of Moldova | 349 | 35.2 | 0.99 |  | 77 | 7.4 | 0.21 |  | 0.21 |
| Romania | 1328 | 39.3 | 1.1 |  | 162 | 4.5 | 0.14 |  | 0.11 |
| Russian Federation | 13407 | 42.8 | 1.2 |  | 2274 | 6.7 | 0.20 |  | 0.16 |
| Rwanda | 416 | 14.4 | 0.42 |  | 178 | 6.1 | 0.18 |  | 0.42 |
| Saint Lucia | 17 | 37.8 | 1.1 |  | 2 | 4.3 | 0.13 |  | 0.11 |
| Samoa | 14 | 43.7 | 1.3 |  | 1 | 2.8 | 0.09 |  | 0.06 |
| Sao Tome and Principe | 7 | 17.2 | 0.49 |  | 3 | 7.4 | 0.23 |  | 0.43 |
| Saudi Arabia | 1104 | 14.2 | 0.41 |  | 221 | 2.7 | 0.08 |  | 0.19 |
| Senegal | 1126 | 32.8 | 0.93 |  | 459 | 13.4 | 0.39 |  | 0.41 |
| Serbia | 441 | 26.1 | 0.76 |  | 59 | 3.3 | 0.10 |  | 0.13 |
| Sierra Leone | 269 | 16.7 | 0.48 |  | 107 | 6.7 | 0.19 |  | 0.4 |
| Singapore | 458 | 37.1 | 1.1 |  | 33 | 2.7 | 0.08 |  | 0.07 |
| Slovakia | 377 | 33.9 | 0.97 |  | 45 | 3.8 | 0.11 |  | 0.11 |
| Slovenia | 117 | 31.6 | 0.92 |  | 8 | 1.9 | 0.06 |  | 0.06 |
| Solomon Islands | 62 | 47.8 | 1.4 |  | 28 | 21.5 | 0.61 |  | 0.45 |
| Somalia | 734 | 27.3 | 0.78 |  | 357 | 13.5 | 0.39 |  | 0.49 |
| South Africa | 4335 | 28.7 | 0.84 |  | 1656 | 11.0 | 0.32 |  | 0.38 |
| South Sudan | 410 | 19.0 | 0.55 |  | 200 | 9.3 | 0.27 |  | 0.49 |
| Spain | 2835 | 35.7 | 1.0 |  | 204 | 2.4 | 0.07 |  | 0.07 |
| Sri Lanka | 432 | 9.8 | 0.29 |  | 116 | 2.6 | 0.08 |  | 0.27 |
| Sudan | 2124 | 24.8 | 0.71 |  | 743 | 8.8 | 0.26 |  | 0.35 |
| Suriname | 52 | 41.8 | 1.2 |  | 6 | 4.9 | 0.14 |  | 0.12 |
| Sweden | 647 | 33.8 | 0.98 |  | 47 | 2.4 | 0.07 |  | 0.07 |
| Switzerland | 514 | 29.7 | 0.86 |  | 28 | 1.5 | 0.05 |  | 0.05 |
| Syrian Arab Republic | 1146 | 24.2 | 0.70 |  | 326 | 6.8 | 0.20 |  | 0.28 |
| Tajikistan | 415 | 19.1 | 0.56 |  | 98 | 4.5 | 0.13 |  | 0.24 |
| Tanzania, United Republic of | 2209 | 19.2 | 0.56 |  | 916 | 8.0 | 0.23 |  | 0.42 |
| Thailand | 3357 | 25.0 | 0.72 |  | 651 | 4.8 | 0.14 |  | 0.19 |
| The Netherlands | 1273 | 41.7 | 1.2 |  | 92 | 3.0 | 0.09 |  | 0.07 |
| The Republic of the Gambia | 187 | 38.2 | 1.1 |  | 90 | 18.3 | 0.51 |  | 0.48 |
| Timor-Leste | 71 | 26.5 | 0.75 |  | 12 | 4.8 | 0.15 |  | 0.18 |
| Togo | 347 | 20.8 | 0.60 |  | 150 | 9.0 | 0.26 |  | 0.43 |
| Total | 406225 | 24.8 | 0.72 |  | 93807 | 5.7 | 0.17 |  | 0.23 |
| Trinidad and Tobago | 113 | 35.2 | 1.0 |  | 29 | 8.8 | 0.26 |  | 0.25 |
| Tunisia | 561 | 19.9 | 0.59 |  | 106 | 3.8 | 0.11 |  | 0.19 |
| Türkiye | 4789 | 26.0 | 0.75 |  | 748 | 4.0 | 0.12 |  | 0.15 |
| Turkmenistan | 375 | 26.0 | 0.75 |  | 118 | 8.0 | 0.24 |  | 0.31 |
| Uganda | 2808 | 31.8 | 0.92 |  | 1240 | 14.2 | 0.42 |  | 0.45 |
| Ukraine | 2875 | 30.0 | 0.87 |  | 591 | 5.9 | 0.18 |  | 0.2 |
| United Arab Emirates | 236 | 10.0 | 0.29 |  | 34 | 1.4 | 0.04 |  | 0.14 |
| United Kingdom | 4771 | 36.0 | 1.0 |  | 475 | 3.5 | 0.10 |  | 0.1 |
| United States of America | 23403 | 35.4 | 1.0 |  | 2054 | 3.1 | 0.09 |  | 0.09 |
| Uruguay | 267 | 39.1 | 1.1 |  | 40 | 5.7 | 0.17 |  | 0.15 |
| Uzbekistan | 1842 | 21.7 | 0.63 |  | 453 | 5.3 | 0.16 |  | 0.24 |
| Vanuatu | 20 | 30.3 | 0.87 |  | 10 | 15.2 | 0.43 |  | 0.5 |
| Venezuela | 1993 | 32.2 | 0.93 |  | 538 | 8.6 | 0.25 |  | 0.27 |
| Viet Nam | 3611 | 15.3 | 0.45 |  | 746 | 3.1 | 0.09 |  | 0.2 |
| Yemen | 1305 | 19.5 | 0.55 |  | 607 | 9.1 | 0.27 |  | 0.47 |
| Zambia | 1047 | 28.9 | 0.84 |  | 352 | 9.8 | 0.29 |  | 0.34 |
| Zimbabwe | 1099 | 34.3 | 1.0 |  | 412 | 12.8 | 0.38 |  | 0.37 |

Note: ASIR=Age-standardized incidence rate; ASMR=Age-standardized mortality rate; HDI=Human development index.
