# Supplementary material for: Female cancers in adolescent and young adult women: global inequities and 2050 burden estimates from GLOBOCAN 2022
Source: Front Oncol. 2026 Aug 6;16:1896950. doi: 10.3389/fonc.2026.1896950 (PMC13489919; doi:10.3389/fonc.2026.1896950)
Supplement: Supplementary file 3 [file SupplementaryFile2.docx]

**Supplementary Table S2. Projected number of female cancer cases among women aged 15–39 years in 2050 and percentage change from 2022, by country**

|  | Incidence | |  | Mortality | |
| --- | --- | --- | --- | --- | --- |
|  | Number of Case in 2050 | Change in number of cases due to demographic change between 2022 and 2050 (Percentage change) |  | Number of Case in 2050 | Change in number of cases due to demographic change between 2022 and 2050 (Percentage change) |
| Afghanistan | 3389 | 1868 (122.81%) |  | 2454 | 1868 (127.65%) |
| Albania | 123 | -69 (-35.94%) |  | -57 | -69 (-25%) |
| Algeria | 4545 | 923 (25.48%) |  | 1781 | 923 (25.52%) |
| Angola | 4339 | 2572 (145.56%) |  | 3184 | 2572 (146.24%) |
| Argentina | 4261 | 36 (0.85%) |  | 887 | 36 (2.7%) |
| Armenia | 65 | -38 (-36.89%) |  | -17 | -38 (-33.33%) |
| Australia | 1963 | 144 (7.92%) |  | 280 | 144 (8.09%) |
| Austria | 441 | -117 (-20.97%) |  | -71 | -117 (-21.74%) |
| Azerbaijan | 492 | -94 (-16.04%) |  | 44 | -94 (-13.77%) |
| Bahamas | 30 | -1 (-3.23%) |  | 5 | -1 (0%) |
| Bahrain | 49 | 1 (2.08%) |  | 8 | 1 (0%) |
| Bangladesh | 6401 | -218 (-3.29%) |  | 2238 | -218 (-2.97%) |
| Barbados | 18 | -5 (-21.74%) |  | -2 | -5 (-33.33%) |
| Belarus | 464 | -163 (-26%) |  | -68 | -163 (-26.32%) |
| Belgium | 791 | -47 (-5.61%) |  | 2 | -47 (-6.12%) |
| Belize | 23 | 2 (9.52%) |  | 8 | 2 (0%) |
| Benin | 712 | 396 (125.32%) |  | 521 | 396 (126.4%) |
| Bhutan | 17 | -3 (-15%) |  | 6 | -3 (-11.11%) |
| Bolivia (Plurinational State of) | 1641 | 441 (36.75%) |  | 686 | 441 (42.86%) |
| Bosnia Herzegovina | 145 | -61 (-29.61%) |  | -36 | -61 (-28%) |
| Botswana | 270 | 83 (44.39%) |  | 152 | 83 (44.93%) |
| Brazil | 11972 | -2762 (-18.75%) |  | 477 | -2762 (-18.46%) |
| Brunei Darussalam | 38 | -5 (-11.63%) |  | -1 | -5 (0%) |
| Bulgaria | 241 | -143 (-37.24%) |  | -77 | -143 (-37.88%) |
| Burkina Faso | 1321 | 742 (128.15%) |  | 1016 | 742 (131.39%) |
| Burundi | 1324 | 727 (121.78%) |  | 983 | 727 (125.78%) |
| CÃ´te d'Ivoire | 3217 | 1684 (109.85%) |  | 2453 | 1684 (109.75%) |
| Cambodia | 872 | -26 (-2.9%) |  | 274 | -26 (-3%) |
| Cameroon | 3213 | 1637 (103.87%) |  | 2372 | 1637 (102.99%) |
| Canada | 2557 | 86 (3.48%) |  | 363 | 86 (3.97%) |
| Cape Verde | 24 | 0 (0%) |  | 8 | 0 (0%) |
| Central African Republic | 564 | 364 (182%) |  | 470 | 364 (185.85%) |
| Chad | 1652 | 1001 (153.76%) |  | 1409 | 1001 (156.62%) |
| Chile | 891 | -195 (-17.96%) |  | -18 | -195 (-16.95%) |
| China | 42521 | -14531 (-25.47%) |  | -7447 | -14531 (-25.23%) |
| Colombia | 3240 | -554 (-14.6%) |  | 94 | -554 (-13.73%) |
| Comoros | 86 | 25 (40.98%) |  | 43 | 25 (44.44%) |
| Congo, Democratic Republic of | 9172 | 5803 (172.25%) |  | 7280 | 5803 (172.51%) |
| Congo, Republic of | 255 | 138 (117.95%) |  | 188 | 138 (118%) |
| Costa Rica | 232 | -51 (-18.02%) |  | 4 | -51 (-18.18%) |
| Croatia | 173 | -81 (-31.89%) |  | -57 | -81 (-29.17%) |
| Cuba | 391 | -149 (-27.59%) |  | -53 | -149 (-26.04%) |
| Cyprus | 97 | -14 (-12.61%) |  | -9 | -14 (0%) |
| Czechia | 616 | -26 (-4.05%) |  | 26 | -26 (-5.77%) |
| Denmark | 392 | 18 (4.81%) |  | 39 | 18 (4.76%) |
| Djibouti | 86 | 23 (36.51%) |  | 53 | 23 (36.67%) |
| Dominican Republic | 1063 | 80 (8.14%) |  | 318 | 80 (10.92%) |
| Ecuador | 1128 | 87 (8.36%) |  | 286 | 87 (11.06%) |
| Egypt | 9936 | 3518 (54.81%) |  | 5268 | 3518 (54.97%) |
| El Salvador | 325 | -71 (-17.93%) |  | 4 | -71 (-13.33%) |
| Equatorial Guinea | 238 | 127 (114.41%) |  | 175 | 127 (116.67%) |
| Eritrea | 367 | 161 (78.16%) |  | 250 | 161 (78.65%) |
| Estonia | 59 | -21 (-26.25%) |  | -13 | -21 (-25%) |
| Eswatini | 219 | 73 (50%) |  | 139 | 73 (53.03%) |
| Ethiopia | 14276 | 6935 (94.47%) |  | 10319 | 6935 (95.63%) |
| Fiji | 140 | 26 (22.81%) |  | 57 | 26 (22.58%) |
| Finland | 231 | -17 (-6.85%) |  | 0 | -17 (0%) |
| France (metropolitan) | 4984 | -266 (-5.07%) |  | 132 | -266 (-4.27%) |
| France, Guadeloupe | 23 | 2 (9.52%) |  | 6 | 2 (0%) |
| France, La RÃ©union | 57 | 13 (29.55%) |  | 20 | 13 (28.57%) |
| France, Martinique | 17 | -2 (-10.53%) |  | 1 | -2 (0%) |
| French Guyana | 20 | 8 (66.67%) |  | 9 | 8 (100%) |
| French Polynesia | 17 | -1 (-5.56%) |  | 2 | -1 (0%) |
| Gabon | 266 | 104 (64.2%) |  | 154 | 104 (64%) |
| Gaza Strip and West Bank | 367 | 160 (77.29%) |  | 211 | 160 (80.39%) |
| Georgia | 150 | -35 (-18.92%) |  | -1 | -35 (-20.59%) |
| Germany | 3475 | -700 (-16.77%) |  | -185 | -700 (-17.09%) |
| Ghana | 3305 | 1435 (76.74%) |  | 2137 | 1435 (78.35%) |
| Greece | 500 | -140 (-21.88%) |  | -91 | -140 (-20.41%) |
| Guam | 13 | 2 (18.18%) |  | 2 | #DIV/0! |
| Guatemala | 1194 | 274 (29.78%) |  | 457 | 274 (33.88%) |
| Guinea | 1889 | 940 (99.05%) |  | 1366 | 940 (101.17%) |
| Guinea-Bissau | 197 | 89 (82.41%) |  | 134 | 89 (86.67%) |
| Guyana | 54 | 5 (10.2%) |  | 16 | 5 (18.18%) |
| Haiti | 672 | 125 (22.85%) |  | 327 | 125 (21.78%) |
| Honduras | 613 | 127 (26.13%) |  | 332 | 127 (23.9%) |
| Hungary | 500 | -130 (-20.63%) |  | -63 | -130 (-22.39%) |
| Iceland | 21 | 0 (0%) |  | 2 | 0 (0%) |
| India | 50109 | 2141 (4.46%) |  | 18095 | 2141 (4.83%) |
| Indonesia | 23867 | 1263 (5.59%) |  | 6907 | 1263 (6.11%) |
| Iran, Islamic Republic of | 4018 | -777 (-16.2%) |  | -3 | -777 (-17.44%) |
| Iraq | 3840 | 1827 (90.76%) |  | 2418 | 1827 (92.89%) |
| Ireland | 329 | 15 (4.78%) |  | 50 | 15 (2.86%) |
| Israel | 753 | 265 (54.3%) |  | 323 | 265 (53.45%) |
| Italy | 2860 | -947 (-24.88%) |  | -669 | -947 (-24.1%) |
| Jamaica | 226 | -152 (-40.21%) |  | -56 | -152 (-38.54%) |
| Japan | 6616 | -2389 (-26.53%) |  | -1873 | -2389 (-26.16%) |
| Jordan | 835 | 231 (38.25%) |  | 379 | 231 (38.51%) |
| Kazakhstan | 1174 | 221 (23.19%) |  | 505 | 221 (22.89%) |
| Kenya | 3867 | 1483 (62.21%) |  | 2394 | 1483 (63.12%) |
| Korea, Democratic People's Republic of | 1066 | -202 (-15.93%) |  | 397 | -202 (-16.03%) |
| Korea, Republic of | 2857 | -1832 (-39.07%) |  | -1581 | -1832 (-36.65%) |
| Kuwait | 116 | 10 (9.43%) |  | 25 | 10 (0%) |
| Kyrgyzstan | 475 | 127 (36.49%) |  | 193 | 127 (37.88%) |
| Lao People's Democratic Republic | 526 | 89 (20.37%) |  | 196 | 89 (24.3%) |
| Latvia | 103 | -43 (-29.45%) |  | -26 | -43 (-29.41%) |
| Lebanon | 253 | -67 (-20.94%) |  | 13 | -67 (-20%) |
| Lesotho | 266 | 77 (40.74%) |  | 165 | 77 (39.77%) |
| Liberia | 705 | 345 (95.83%) |  | 495 | 345 (98.67%) |
| Libya | 333 | 18 (5.71%) |  | 89 | 18 (5.63%) |
| Lithuania | 135 | -35 (-20.59%) |  | -6 | -35 (-20.69%) |
| Luxembourg | 47 | -2 (-4.08%) |  | 2 | -2 (0%) |
| Madagascar | 3590 | 1749 (95%) |  | 2439 | 1749 (97.97%) |
| Malawi | 4844 | 2544 (110.61%) |  | 3753 | 2544 (111.66%) |
| Malaysia | 2344 | -131 (-5.29%) |  | 479 | -131 (-4.92%) |
| Maldives | 39 | -6 (-13.33%) |  | 2 | -6 (-12.5%) |
| Mali | 3395 | 2149 (172.47%) |  | 2781 | 2149 (172.47%) |
| Malta | 32 | -7 (-17.95%) |  | -4 | -7 (0%) |
| Mauritania | 539 | 266 (97.44%) |  | 377 | 266 (98.2%) |
| Mauritius | 52 | -17 (-24.64%) |  | -4 | -17 (-23.08%) |
| Mexico | 7556 | -556 (-6.85%) |  | 879 | -556 (-4.32%) |
| Mongolia | 151 | 33 (27.97%) |  | 60 | 33 (29.63%) |
| Montenegro | 20 | -5 (-20%) |  | 2 | -5 (-28.57%) |
| Morocco | 2711 | 177 (6.99%) |  | 693 | 177 (6.78%) |
| Mozambique | 6379 | 3575 (127.5%) |  | 5153 | 3575 (126.93%) |
| Myanmar | 3564 | -145 (-3.91%) |  | 1073 | -145 (-2.79%) |
| Namibia | 314 | 105 (50.24%) |  | 177 | 105 (52.78%) |
| Nepal | 1116 | 10 (0.9%) |  | 419 | 10 (1.96%) |
| New Caledonia | 28 | 3 (12%) |  | 8 | 3 (0%) |
| New Zealand | 375 | 31 (9.01%) |  | 68 | 31 (10.81%) |
| Nicaragua | 356 | 54 (17.88%) |  | 145 | 54 (18.68%) |
| Niger | 3704 | 2514 (211.26%) |  | 3122 | 2514 (210.53%) |
| Nigeria | 15208 | 8021 (111.6%) |  | 10908 | 8021 (112.89%) |
| North Macedonia | 111 | -55 (-33.13%) |  | -32 | -55 (-30.43%) |
| Norway | 422 | 17 (4.2%) |  | 35 | 17 (5.56%) |
| Oman | 287 | 73 (34.11%) |  | 137 | 73 (32.81%) |
| Pakistan | 15320 | 6208 (68.13%) |  | 8741 | 6208 (69.92%) |
| Panama | 398 | 61 (18.1%) |  | 118 | 61 (21.05%) |
| Papua New Guinea | 1355 | 562 (70.87%) |  | 919 | 562 (71.43%) |
| Paraguay | 909 | 22 (2.48%) |  | 188 | 22 (3.61%) |
| Peru | 3027 | 182 (6.4%) |  | 660 | 182 (7.74%) |
| Philippines | 9693 | 2424 (33.35%) |  | 3904 | 2424 (34.39%) |
| Poland | 1606 | -737 (-31.46%) |  | -452 | -737 (-34.04%) |
| Portugal | 707 | -199 (-21.96%) |  | -119 | -199 (-22.5%) |
| Puerto Rico | 121 | -26 (-17.69%) |  | -3 | -26 (-13.04%) |
| Qatar | 94 | 24 (34.29%) |  | 36 | 24 (25%) |
| Republic of Moldova | 199 | -150 (-42.98%) |  | -73 | -150 (-42.86%) |
| Romania | 1097 | -231 (-17.39%) |  | -69 | -231 (-19.14%) |
| Russian Federation | 10408 | -2999 (-22.37%) |  | -725 | -2999 (-23.57%) |
| Rwanda | 713 | 297 (71.39%) |  | 475 | 297 (72.47%) |
| Saint Lucia | 11 | -6 (-35.29%) |  | -4 | -6 (-50%) |
| Samoa | 28 | 14 (100%) |  | 15 | 14 (100%) |
| Sao Tome and Principe | 14 | 7 (100%) |  | 10 | 7 (100%) |
| Saudi Arabia | 1279 | 175 (15.85%) |  | 396 | 175 (15.38%) |
| Senegal | 2225 | 1099 (97.6%) |  | 1558 | 1099 (98.47%) |
| Serbia | 230 | -211 (-47.85%) |  | -152 | -211 (-47.46%) |
| Sierra Leone | 508 | 239 (88.85%) |  | 346 | 239 (93.46%) |
| Singapore | 343 | -115 (-25.11%) |  | -82 | -115 (-24.24%) |
| Slovakia | 280 | -97 (-25.73%) |  | -52 | -97 (-26.67%) |
| Slovenia | 101 | -16 (-13.68%) |  | -8 | -16 (-12.5%) |
| Solomon Islands | 122 | 60 (96.77%) |  | 88 | 60 (96.43%) |
| Somalia | 2032 | 1298 (176.84%) |  | 1655 | 1298 (180.67%) |
| South Africa | 4672 | 337 (7.77%) |  | 1993 | 337 (7.67%) |
| South Sudan | 794 | 384 (93.66%) |  | 584 | 384 (97.5%) |
| Spain | 2294 | -541 (-19.08%) |  | -337 | -541 (-19.61%) |
| Sri Lanka | 393 | -39 (-9.03%) |  | 77 | -39 (-8.62%) |
| Sudan | 4454 | 2330 (109.7%) |  | 3073 | 2330 (112.79%) |
| Suriname | 61 | 9 (17.31%) |  | 15 | 9 (16.67%) |
| Sweden | 709 | 62 (9.58%) |  | 109 | 62 (8.51%) |
| Switzerland | 483 | -31 (-6.03%) |  | -3 | -31 (-3.57%) |
| Syrian Arab Republic | 1652 | 506 (44.15%) |  | 832 | 506 (43.87%) |
| TÃ¼rkiye | 4625 | -164 (-3.42%) |  | 584 | -164 (-2.94%) |
| Tajikistan | 628 | 213 (51.33%) |  | 311 | 213 (53.06%) |
| Tanzania, United Republic of | 5209 | 3000 (135.81%) |  | 3916 | 3000 (135.81%) |
| Thailand | 2602 | -755 (-22.49%) |  | -104 | -755 (-21.2%) |
| The Netherlands | 1177 | -96 (-7.54%) |  | -4 | -96 (-7.61%) |
| The Republic of the Gambia | 398 | 211 (112.83%) |  | 301 | 211 (111.11%) |
| Timor-Leste | 100 | 29 (40.85%) |  | 41 | 29 (58.33%) |
| Togo | 672 | 325 (93.66%) |  | 475 | 325 (94%) |
| Trinidad and Tobago | 92 | -21 (-18.58%) |  | 8 | -21 (-17.24%) |
| Tunisia | 585 | 24 (4.28%) |  | 130 | 24 (4.72%) |
| Turkmenistan | 478 | 103 (27.47%) |  | 221 | 103 (28.81%) |
| Uganda | 6329 | 3521 (125.39%) |  | 4761 | 3521 (128.55%) |
| Ukraine | 1732 | -1143 (-39.76%) |  | -552 | -1143 (-39.26%) |
| United Arab Emirates | 228 | -8 (-3.39%) |  | 26 | -8 (-2.94%) |
| United Kingdom | 4481 | -290 (-6.08%) |  | 185 | -290 (-5.47%) |
| United States of America | 23578 | 175 (0.75%) |  | 2229 | 175 (1.22%) |
| Uruguay | 229 | -38 (-14.23%) |  | 2 | -38 (-12.5%) |
| Uzbekistan | 2098 | 256 (13.9%) |  | 709 | 256 (13.02%) |
| Vanuatu | 37 | 17 (85%) |  | 27 | 17 (80%) |
| Venezuela | 2322 | 329 (16.51%) |  | 867 | 329 (17.29%) |
| Viet Nam | 2976 | -635 (-17.59%) |  | 111 | -635 (-17.16%) |
| Yemen | 2377 | 1072 (82.15%) |  | 1679 | 1072 (85.67%) |
| Zambia | 2370 | 1323 (126.36%) |  | 1675 | 1323 (127.56%) |
| Zimbabwe | 2118 | 1019 (92.72%) |  | 1431 | 1019 (93.2%) |
