# Supplementary material for: Female cancers in adolescent and young adult women: global inequities and 2050 burden estimates from GLOBOCAN 2022
Source: Front Oncol. 2026 Aug 6;16:1896950. doi: 10.3389/fonc.2026.1896950 (PMC13489919; doi:10.3389/fonc.2026.1896950)
Supplement: Supplementary file 4 [file SupplementaryFile3.docx]

**Supplementary Table S3. Projected numbers of breast, ovarian, and cervical cancer cases among women aged 15–39 years by 2050, with percentage changes from 2022, according to cancer type and HDI level**

| Cancer type | Incidence | |  | Mortality | |
| --- | --- | --- | --- | --- | --- |
|  | Number of Case in 2050 | Change in number of cases due to demographic changes between 2022 and 2050 (Percentage change) |  | Number of Case in 2050 | Change in number of cases due to demographic changes between 2022 and 2050 (Percentage change) |
| Breast |  |  |  |  |  |
| Very high HDI countries | 67233 | -7410 (-9.93%) |  | 6347 | -677 (-9.64%) |
| High HDI countries | 75904 | -11292 (-12.95%) |  | 11726 | -1678 (-12.52%) |
| Medium HDI countries | 60016 | 8343 (16.15%) |  | 17737 | 2500 (16.41%) |
| Low HDI countries | 67077 | 34865 (108.24%) |  | 27074 | 14112 (108.87%) |
| Ovary |  |  |  |  |  |
| Very high HDI countries | 7134 | -809 (-10.19%) |  | 1252 | -136 (-9.8%) |
| High HDI countries | 12065 | -1970 (-14.04%) |  | 2869 | -444 (-13.4%) |
| Medium HDI countries | 12592 | 1539 (13.92%) |  | 3914 | 483 (14.08%) |
| Low HDI countries | 9472 | 4810 (103.17%) |  | 3475 | 1763 (102.98%) |
| Cervix uteri |  |  |  |  |  |
| Very high HDI countries | 20817 | -2412 (-10.38%) |  | 3623 | -400 (-9.94%) |
| High HDI countries | 32786 | -5009 (-13.25%) |  | 8315 | -1237 (-12.95%) |
| Medium HDI countries | 33644 | 4808 (16.67%) |  | 13462 | 1994 (17.39%) |
| Low HDI countries | 33137 | 17310 (109.37%) |  | 15837 | 8313 (110.49%) |

Note: HDI=Human development index.
